# Dysregulated transcription across diverse cancer types reveals the importance of RNA-binding protein in carcinogenesis

**DOI:** 10.1186/1471-2164-16-S7-S5

**Published:** 2015-06-11

**Authors:** Jing Wang, Qi Liu, Yu Shyr

**Affiliations:** 1 Center for Quantitative Sciences, Vanderbilt University School of Medicine, Nashville, TN 37232, USA; 2 Department of Biomedical Informatics, Vanderbilt University School of Medicine, Nashville, TN 37232, USA; 3 Department of Cancer Biology, Vanderbilt University School of Medicine, Nashville, TN 37232, USA; 4 Department of Biostatistics, Vanderbilt University School of Medicine, Nashville, TN 37232, USA

**Keywords:** Dysregulated transcription, Pan-cancer, RBP, Tissue-specific genes, Cancer-related genes

## Abstract

**Background:**

It is well known that carcinogenesis is in part dictated by dysregulated transcription events and signal pathways. Large-scale transcriptional profiling studies in each cancer type have reported aberrant gene expression associated with cancer development. However, common and specific patterns altered across cancer types, especially the contribution of transcriptional and post-transcriptional regulators, are rarely explored.

**Results:**

Using transcriptional profiles from matched tumor and normal samples in the Cancer Genome Atlas pan-cancer dataset, we performed a comprehensive analysis on the altered expression across 9 cancer types, focusing on transcriptional and post-transcriptional regulators and cancer-related genes. As we expected, the transcription of cancer-related genes was significantly deregulated in tumor vs. normal across all cancer types. Surprisingly, the expression of RNA-binding proteins (RBPs), master regulators of post-transcriptional gene expression, was also significantly changed across most studied cancer types. Although the expression of RBPs was not as strongly deregulated as cancer-related genes, their direct interaction partners are enriched by cancer-related genes, suggesting the cascade regulation effect of RBPs. Integrating genetic and epigenetic profiles found that deregulated RBPs were frequently caused by genetic rather than epigenetic alterations. Furthermore, tissue-specific genes were under-expressed in tumor vs. normal across all cancer types except prostate cancer.

**Conclusions:**

Dysregulated transcription across cancer types reveals the importance of RBPs in carcinogenesis. The aberrant expression of RBPs is caused by genetic alterations and spreads their effect to cancer-related genes. In addition, disruption of tissue-specific genes contributes to the corresponding cancer pathology.

## Background

Cancer development is characterized by uncontrolled cell proliferation, which is in part due to expression alteration of genes which regulate cell growth and differentiation, such as the improper over-expression of oncogenes, or the under-expression or disabling of tumor suppressor genes [1]. Comparative analysis of expression alterations between tumor and matched normal samples in each individual cancer type has identified many transcriptional and post-transcriptional regulators associated with carcinogenesis [2-13]. For instance, compared to normal mucosa, transcription factor (TF) NRF2 was found over-expressed in head and neck squamous cell carcinoma [2]. Using transcriptional data of 17 adenomas and paired samples of normal mucosa, the transcription-regulating network of colorectal adenomas is characterized by significantly altered expression of over 250 TF genes [13]. Compared to TFs, expression alteration of RNA-binding proteins (RBPs), master regulators at the post-transcriptional level, was less studied but deregulated transcriptions of several RBPs also have been reported to play a critical role in human cancers [9-12]. For example, QKI was frequently down-regulated in lung cancer, and QKI-5 inhibited the proliferation and transformation of lung cancer cells [14]. Transcription profiling analysis of RBPs uncovered their aberrant function associated with prostate adenocarcinoma, colon adenocarcinoma, and breast carcinoma as well [9,12,15]. Additionally, aberrant expression of microRNAs (miRNAs) and long non-coding RNAs' (lncRNAs') also led to cancer development [16-20]. However, common and specific patterns altered across different cancer types, especially the contribution of transcriptional and post-transcriptional regulators, are rarely known.

Large-scale genomics projects, such as the Cancer Genome Atlas (TCGA), provided various omics data for thousands of tumors with matched normal samples, including genetic, epigenetic, transcriptomics and proteomics data [21], which gave us a great opportunity to perform pan-cancer studies for understanding the common and specific profiles across multiple cancer types. Recently, landscapes of somatic mutation, copy number alterations and oncogenic signatures across major cancer types have been studied [22-24], as well as microRNA-target interaction and functional proteomics data analysis [25,26]. However, as far as we know, comparative analysis of expression alterations of transcriptional and post-transcriptional regulators across cancer types has never been explored.

In this study, we characterized the expression perturbation of TFs, RBPs, lncRNAs, cancer related genes (allOnco) and other genes on 522 matched tumor and normal tissue pairs across 9 cancer types. We first analyzed the differential expression between matched tumor and normal for each type of gene sets across all studied cancer types, and compared their amplitude of alterations. Then we integrated genetic and epigenetic data and protein-protein interaction network (PPI) to explain the upstream cause and downstream effect of dysregulated transcription. Finally we compared expression changes of tissue-specific genes with non-specific ones and investigated the consistent pathway changes across different cancer types.

## Results and discussion

### Expression alteration of RBPs contributes to cancer development

**Figure 1:**
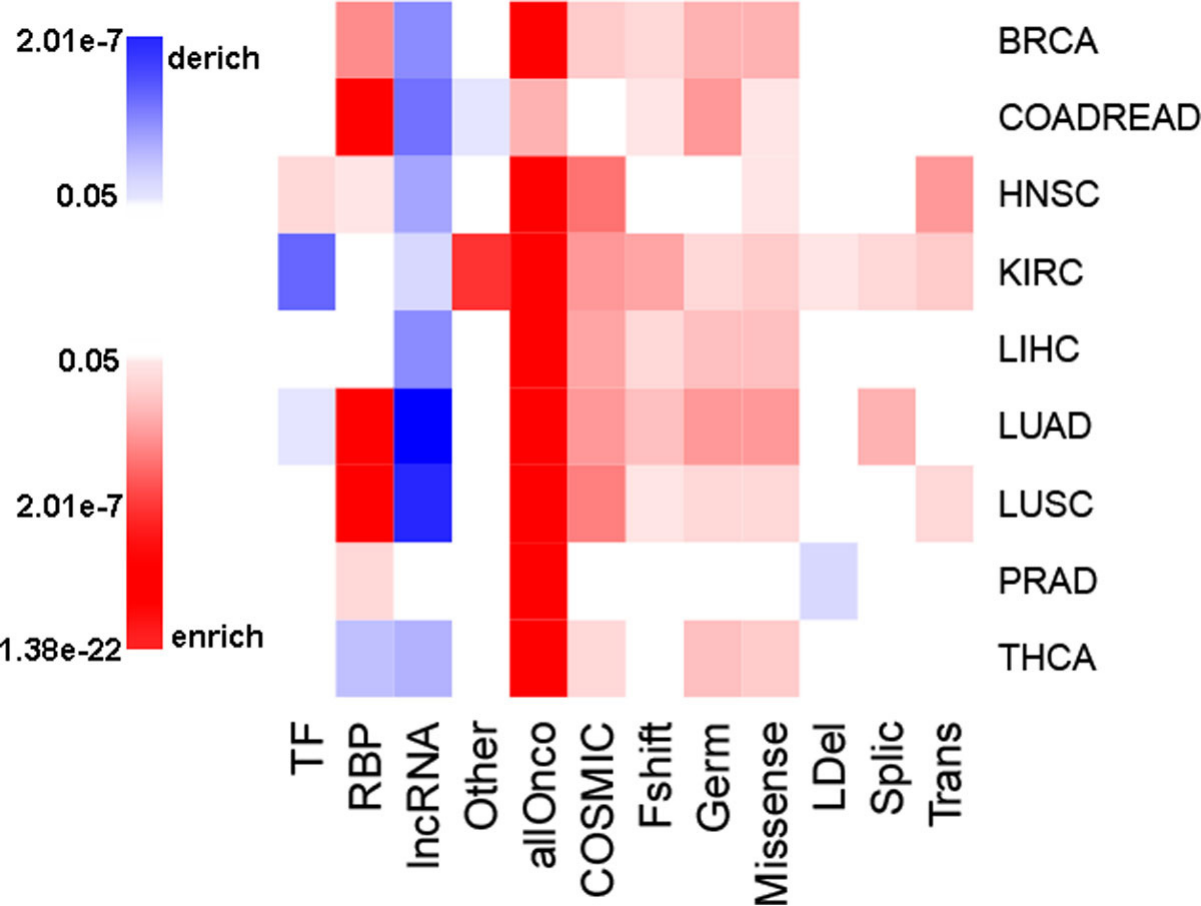
**Differential expression of different types of genes across cancers**. The color represents p-values of enrichment analysis, which ranges from blue, which corresponds to derichment, to red at enrichment. The p-values that greater than 0.05 are colored as white. Fshift refers to frameshift, and Germ, LDel, Trans and Splic refers to germline, large deletions, translocations and splicing mutations respectively.

Thousands of differentially expressed genes were detected in each individual cancer type. The number of TFs, RBPs, lncRNAs, cancer related genes (allOnco), as well as other genes whose expressions were significantly changed was shown in Table 1. As we expected, allOnco were enriched in the differently expressed genes across all cancer types (Figure 1, Table 1), which was supported by many previous reports [27-31]. Mutations in COSMIC with frameshift, germline and missense mutations were also significantly changed across most cancer types, while those with large deletions, translocations and splicing mutations were not (Figure 1, Table 1) [32,33].

**Table 1:** Number and significance of differently expressed genes.

CancerType	BRCA	COADREAD	HNSC	KIRC	LIHC	LUAD	LUSC	PRAD	THCA
**Diff-Expressed**	8556	4139	2226	8210	3385	6422	7981	3133	5727
**TF**	772	369	233*	667	309	556	704	291	556
**RBP**	383***	244***	102*	303	148	354***	420***	144*	192
**lncRNA**	83	30	14	89	24	46	67	33	54
**Other**	7352	3522	1890	7175***	2920	5506	6838	2680	4943
**allOnco**	1020*	445*	313***	960***	429***	752***	924***	385***	661***
**COSMIC**	217*	101	77***	218**	101**	177**	218***	67	150*
**Fshift**	55*	29*	17	58**	26*	47*	50*	15	34
**Germ**	44*	28**	11	40*	22*	38**	39*	14	32*
**Missense**	86*	42*	26*	81*	40*	71**	77*	25	59*
**LDel**	18	11	6	20*	9	15	12	1	13
**Splic**	31	16	10	36*	16	32*	30	8	19
**Trans**	127	55	48**	131*	53	99	124*	38	88

P < 0.05 *, P < 0.001 **, P < 0.0001 ***

Fshift: frameshift, LDel: large deletions, Trans: translocations, Splic: splicing mutations, and Germ: germline.

Surprisingly, RBPs were significantly changed in 6 of the 9 cancer types. Marginal significance was observed in PRAD (p-value = 0.01) and HNSC (p-value = 0.04), while highly statistical significance was detected in COADREAD, LUAD and LUSC (p-value = 3.82e-13, 1e-15 and 4.04e-16 respectively) (Figure 1). Consistent expression alterations of RBPs across different cancer types suggested that they play an important role in carcinogenesis. Compared to RBPs, TFs only showed marginally significant enrichment in HNSC, possibly due to the fact that the activity changes of TF are at the protein level which cannot be reflected at the transcription level (Figure 1). lncRNAs were significantly depleted across all cancer types (Figure 1), which are possibly biased because only 264 of 9227 lncRNAs were included in the standardized mRNA-Seq data in Firehose (see Materials and Methods). Additionally the expression level of lncRNAs is especially low compared to other regulation factors [15].

**Figure 2:**
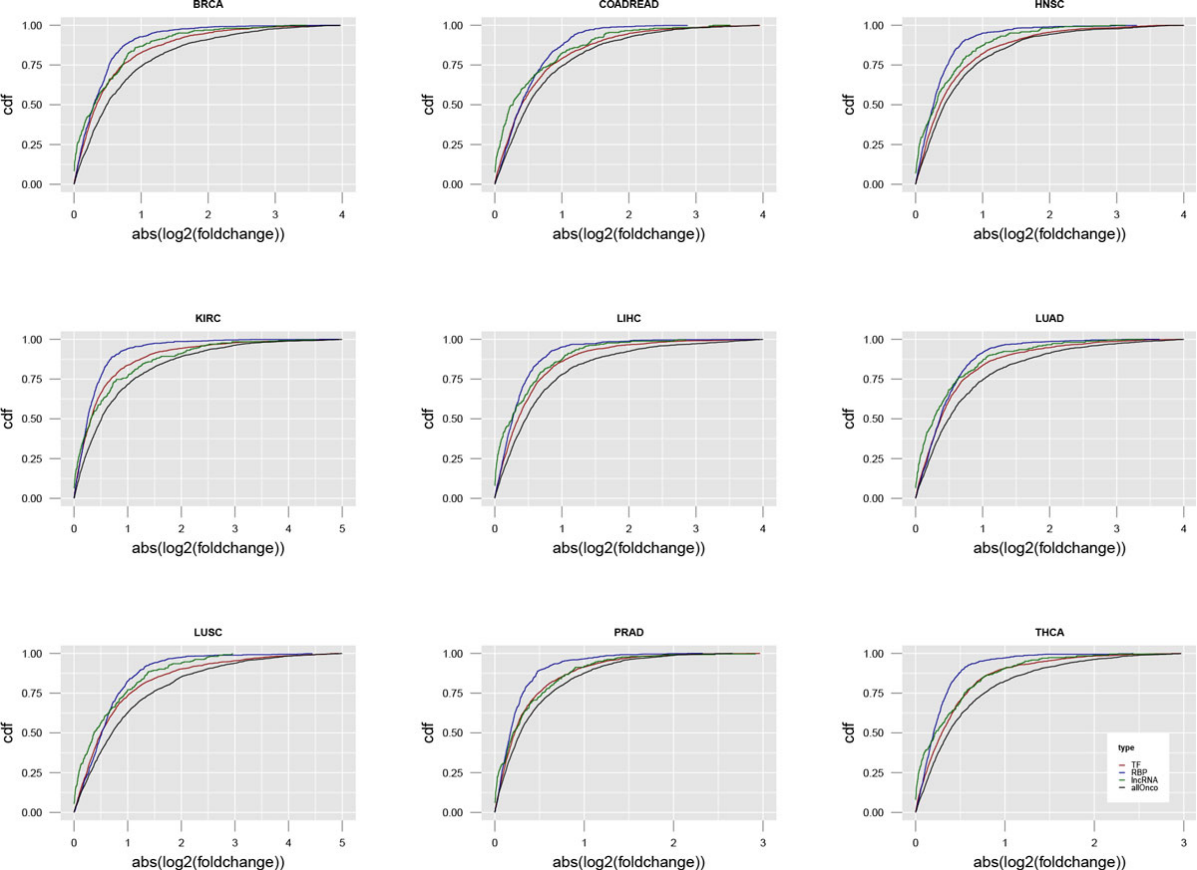
**Comparison of expression alterations between RBPs and other kinds of genes**. Each of the 9 plots illustrate the cumulative distribution function (cdf) of expression change in tumor vs. normal across BRCA, COADREAD, HNSC, KIRC, LIHC, LUAD, LUSC, PRAD and THCA. The x-axis is the absolute value of log2 transformation of fold change.

**Figure 3:**
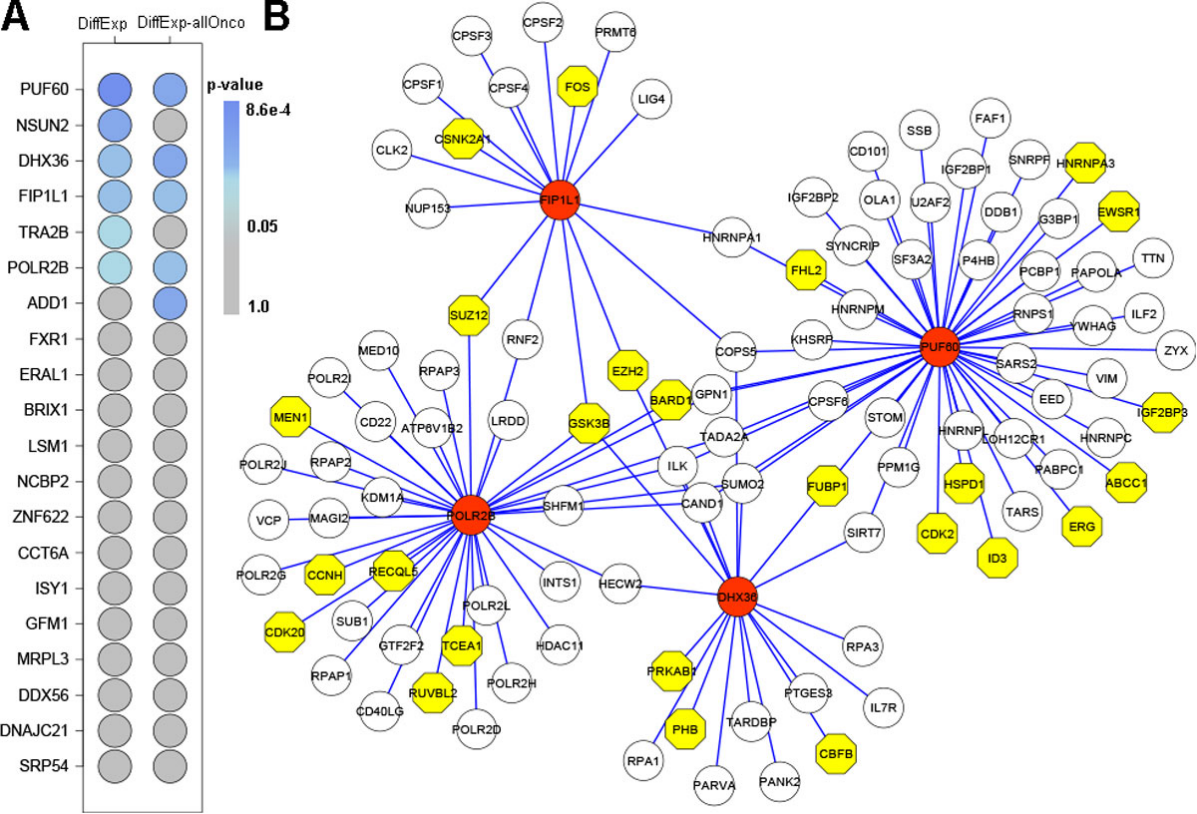
**Network of RBP-target interactions in LUSC**. (A) Enrichment level of differentially expressed genes and allOnco in the targets of top 20 RBPs with highest fold change in LUSC. (B) PPI network comprising interactions between RBPs and differentially expressed targets in LUSC. The RBPs are those in top 20 and both differentially expressed genes and allOnco enriched in their interaction targets. RBPs are color coded as red, and their target allOnco are color coded as yellow.

To further analysis to which extent expression levels were altered in tumor vs. normal, we compared the amplitude of alterations between TFs, RBPs, lncRNAs and allOnco across cancer types. Similar patterns were observed across all cancer types (Figure 2), where cancer-related genes changed most and RBPs had the smallest alterations. Since RBPs and cancer-related genes were both significantly changed across most cancer types, we tried to explore the potential relationships between them. As a result, we found the occurrence of differentially expressed genes and cancer-related genes in the interacting proteins of the RBP of interest. Among the top 20 RBPs changed mostly in tumor compared to normal tissue in LUSC, *PUF60, DHX36, FIP1L1 *and *POLR2B *were identified that both differentially expressed genes and allOnco were enriched in their directly interacting targets (Figure 3). *PUF60, NOP2 *and *PABPC1 *were also identified in LUAD (Additional file 1). It is known that *PUF60 *involved in apoptosis and transcription regulation and isoform 6 may contribute to tumor progression by enabling increased MYC expression and greater resistance to apoptosis in tumors than in normal cells [34,35]. Moreover, there were 7 allOnco genes (*BARD1, ERG, FHL2, FUBP1, HSPD1, ID3 *and *IGF2BP3*) that interacted directly with *PUF60 *and were also differentially expressed in both LUAD and LUSC (Figure 3 and Additional file 1). *BARD1 *acts as tumor suppressors, and plays a central role in the control of the cell cycle and death (apoptosis) and regulates cell division [36-38]. Zhang YQ *et al*. reported that one isoform of *BARD1 *was specifically upregulated in tumors of non-small cell lung cancer [39]. *IGF2BP3, ERG, FUBP1 *and *FHL2 *were also reported being over-expressed/ de-expressed in cancer [40-45]. These results revealed that expression alteration of RBPs might spread their effect to allOnco, which drives the cancer development.

**Figure 4:**
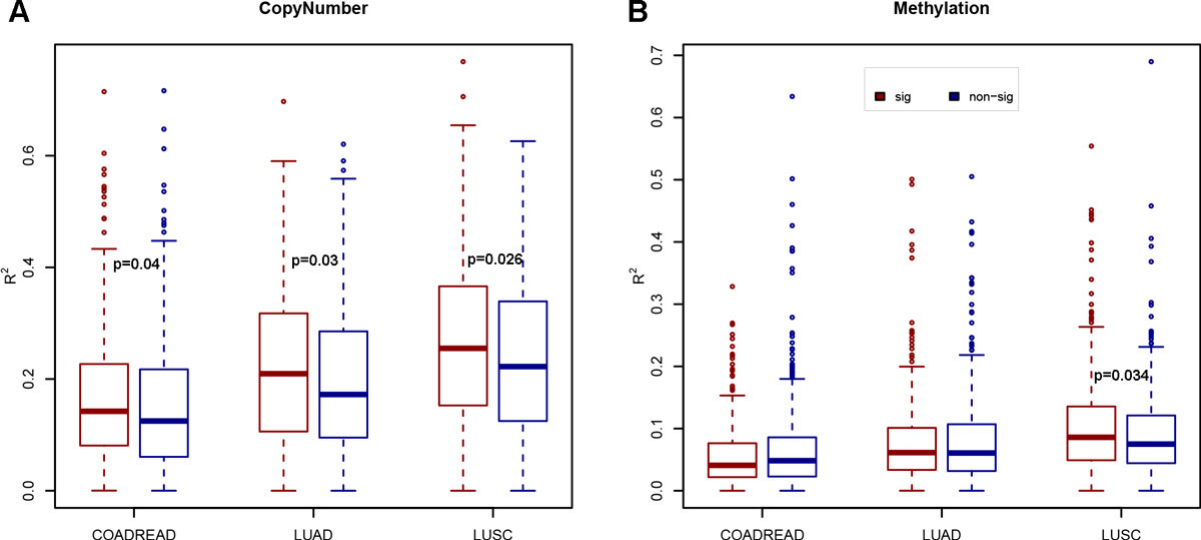
**Genetic and epigenetic alterations regulating RBPs**. For the differentially expressed and other RBPs in COADREAD, LUAD and LUSC, we estimated the extent to which changes in copy number (A) and methylation (B) could explain the variation in their expression (R^2^).

To explore the upstream regulators that changed the RBPs expression, we first compared the correlations of DNA copy number alterations with expression changes between differentially expressed RBPs and non-differentially expressed RBPs. The correlation was represented as R^2 ^to see the extent to which the variation in RBP expression can be explained by DNA copy number alterations at the RBP locus. As aforementioned, RBPs were remarkably enriched in differentially expressed genes in COAD, LUAD and LUSC. Here the results showed that these differentially expressed RBPs had a higher R^2 ^than non-differentially expressed RBPs in all these three cancer types (p-value < 0.05, Wilcoxon rank-sum test; Figure 4A) We also studied the effect of DNA copy number alteration for other 6 studied cancer types and no significance was observed (Additional file 2). We then analyzed the influence of DNA methylation on RBP expression alterations using similar statistical methods, and results showed that DNA methylation changes were not significantly associated with RBP expression alterations (Figure 4B, Additional file 2). These results suggested that the aberrant expression of RBPs was caused by genetic alterations rather than epigenetic alterations.

### Tissue-specific genes lost function in tumor

**Figure 5:**
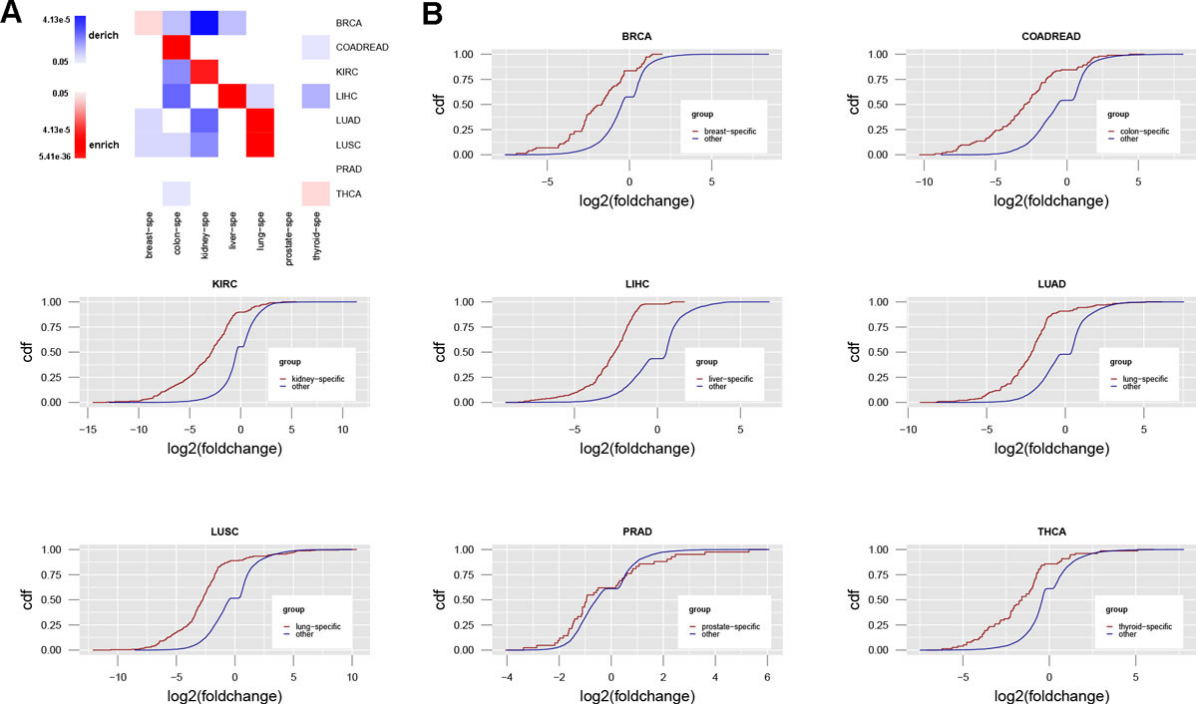
**Tissue-specific genes lost function in tissue associate cancers**. (A) Enrichment p-values of tissue-specific genes in differentially expressed gene set across studied cancer types. (B) Comparison of expression fold change of tissue-specific genes with other genes expressed differently across 8 cancer types.

It is known that some genes are overexpressed in one or several tissues [46], and these tissue-specific genes are highly correlated with tissue-specific diseases [47]. We sought to better understand the role of tissue-specific genes in pan-cancer. We investigated the profile of 2570 specific genes for 7 tissues from PaGenBase (Methods and Materials) [48], and compared their expression in tumor with that in normal. We observed that tissue-specific genes were more likely to be significantly changed in their corresponding cancer type except prostate-specific genes (Figure 5A). For instance, kidney-specific genes were significantly enriched in differentially expressed genes of KIRC (p-value = 3.47e-5) and lung-specific genes showed enrichment in differentially expressed genes for both LUAD and LUSC (p-value = 4.72e-7 and 3.4e-10). However, prostate-specific genes didn't show significance in PRAD, which might be due to the reason that most of the PRAD data were collected from patients in the late stage. Furthermore, most tissue-specific genes were under-expressed in tumor vs. normal across all cancer types except for PRAD (Figure 5B). These results suggested tissue-specific genes generally lost their function in cancer and that defects of tissue-specific genes leads to cancer pathology.

### Functional similarity across different cancer types

**Figure 6:**
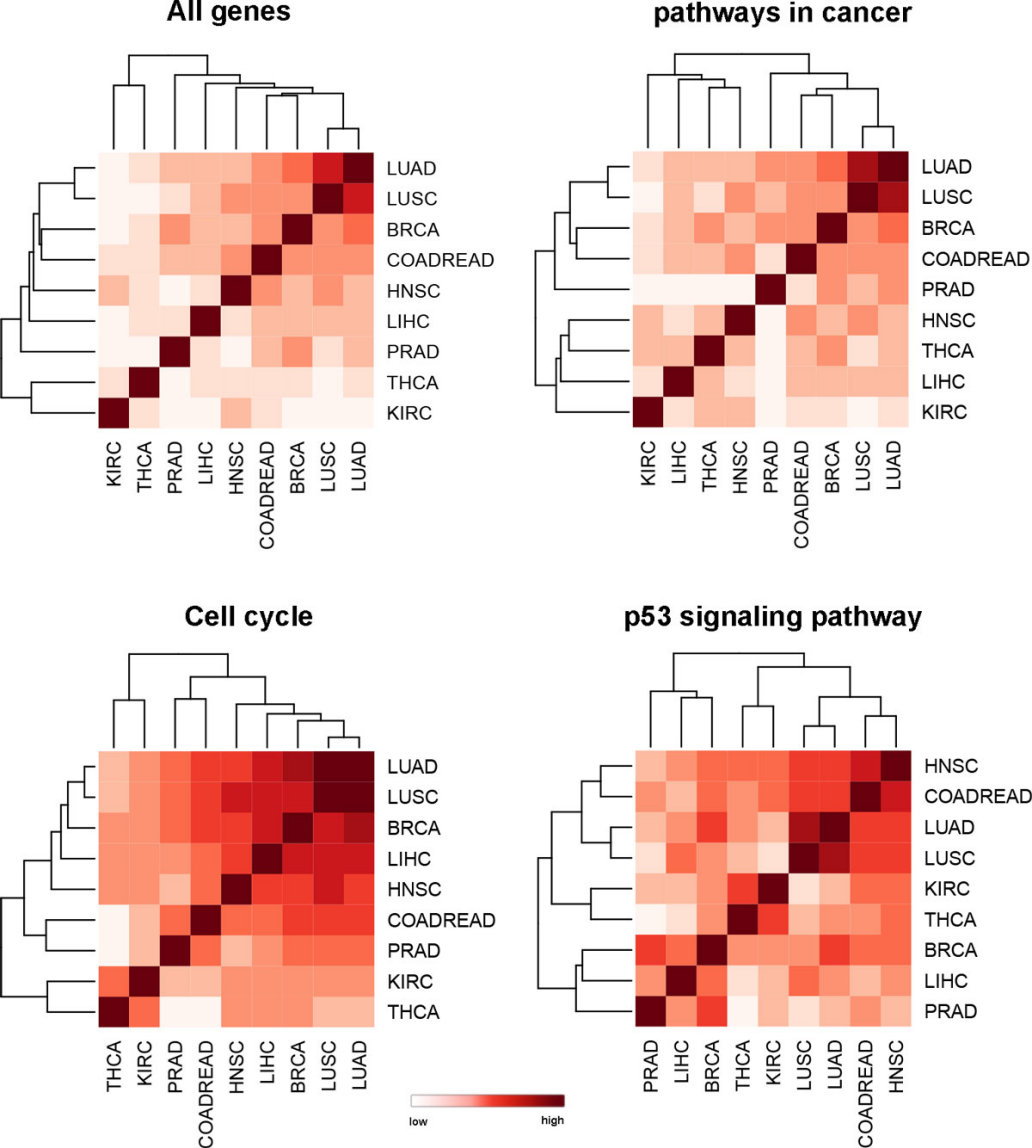
**Cancer type clustering by expression perturbation pattern of genes involved in different pathways**. The four plots present clustering results by all expressed genes, genes involved in pathways in cancer, cell cycle and p53 signaling pathways respectively. The distance of two cancer types is measured by correlation of their expression fold change of involved genes.

Pathways play key roles in genomic studies, and facilitate the understanding of molecular mechanisms behind specific cancers [49]. We estimated the similarity of cancer types not only based on all differentially expressed genes, but also based on the expression alterations of cancer-related pathways from KEGG [50,51], including pathways in cancer, cell cycle and the p53 signaling pathway (Figure 6). As we expected, LUAD and LUSC were clustered together in all kinds of clustering, illustrating their close relationship. Surprisingly, BRCA, LIHC, LUAD and LUSC clustered together in the cell cycle pathway, indicating the cell cycle are disrupted similarly in these cancer types [52-54]. Meanwhile, HNSC and COADREAD were more close to each other in the p53 signaling pathway, which can be explained by the similar regulation role of p53 on these two types of cancer [55,56].

## Conclusions

Dysregulated transcription of RBPs plays an important role in cancer development. The aberrant expression of RBPs is caused by genetic alterations and spreads their effect to cancer-related genes. In addition, disruption of tissue-specific genes contributes to the corresponding cancer pathology.

## Methods and materials

### Genetic, epigenetic and transcriptomics data for 9 cancer types

The mRNA-Seq data of 522 matched tumor and adjacent normal samples for 9 cancer types, the copy number alterations, and the DNA methylation data were downloaded from Firehose developed by the Broad GDAC (https://confluence.broadinstitute.org/display/GDAC/Dashboard-Stddata). The nine cancer types are BRCA (Breast cancer carcinoma), COADREAD (colon/ rectum adenocarcinoma), HNSC (head and neck squamous cell carcinoma), LUAD (lung adenocarcinoma), KIRC (kidney renal clear cell carcinoma), LIHC (liver hepatocellular carcinoma), LUSC (lung squamous cell carcinoma), THCA (thyroid carcinoma) and PRAD (prostate adenocarcinoma). There are 111 paired samples for BRCA, 32 for COADREAD, 41 for HNSC, 72 for KIRC, 57 for LUAD, 59 pairs for THCA and 50 for each type of LUSC, LIHC and PRAD, respectively.

### Different gene sets

1889 TFs were collected from TRANSFAC [57], and 799 experimentally characterized RBPs were obtained from a recent publication dissecting transcriptional profiles of RNA-binding protein in cancer [15]. Over 9,000 lncRNAs were downloaded from Genecode [58,59], but only 264 of these were included in mRNA-Seq data from the Broad GDAC standardized data packages. A comprehensive list of 2102 cancer related genes (allOnco), which is a non-redundant union of 8 studies [33,60-64], was downloaded from Bushman Lab (http://www.bushmanlab.org/links/genelists). About 2570 tissue-specific genes were collected from PaGenBase, which defines genes to be tissue-specific if they are dominantly expressed in one tissue. There are 145 breast-specific, 364 colon-specific, 480 kidney-specific, 628 liver-specific, 643 lung-specific, 263 prostate-specific and 227 thyroid-specific genes, respectively [48]. Different types of somatic mutations, including frameshift mutations, germline mutations, missense mutations, large deletions, splicing mutations and translocations were collected from COSMIC [32].

### Statistical evaluation of differential expression

Paired t-test was used to detect differentially expressed genes between matched tumor and normal tissue pairs. Bonferroni method was used to adjust p-values for multiple testing. Hypergeometric test was used to evaluate the enrichment of different types of genes in the set of differentially expressed genes. All statistical tests in this study were implemented in R (version 3.0.3) [65].

Cytoscape was used to visualize Protein-protein interaction data from PINA2 [66]. Only genes interacting with the RBP of interest were shown in Figure 3 and Additional file 1.

Pairwise Spearman correlations were calculated between the copy number alterations/DNA methylation alterations and gene expression changes for differentially and non-differentially expressed RBPs. The statistical difference of the correlation coefficients were assessed by Wilcoxon Rank Sum test [67].

### Clustering by biological pathways

KEGG pathways are wiring diagrams of molecular interactions, reactions, and relations, and mainly used for biological interpretation of higher-level systemic functions. Different cancers may have consistent changes in some cancer related pathways. To find those pathways similarly altered across different cancers, we performed hierarchical clustering under some specific pathways, including cell cycle, cell proliferation, pathways in cancer and etc. The distance matrix was calculated by Spearman correlation coefficient of expression alteration between different cancer types.

## Competing interests

The authors declare that they have no competing interests.

## Authors' contributions

JW carried out the analysis and drafted the manuscript. QL conceived of the study, guided the analysis, and revised the manuscript. YS supervised the research. All authors read and approved the final manuscript.

## Supplementary Material

Additional file 1 **Network of RBP-target interactions in LUAD**. (A) Enrichment level of differentially expressed genes and allOnco in the targets of top 20 RBPs with highest fold change in LUAD. (B) PPI network comprising interactions between RBPs and differentially expressed targets in LUAD. The RBPs are those in top 20 and both differentially expressed genes and allOnco enriched in their interaction targets. RBPs are color coded as red, and their target allOnco are color coded as yellow. Click here for file

Additional file 2 Genetic and epigenetic alterations regulating RBPs. For the differentially expressed and other RBPs in cancer types of BRAD, HNSC, KIRC, LIHC, PRAD and THCA, we estimated the extent to which changes in copy number and methylation could explain the variation in their expression (R^2^). Click here for file

## Declarations

The publication charges for this article have been funded by the corresponding authors.

This article has been published as part of *BMC Genomics *Volume 16 Supplement 7, 2015: Selected articles from The International Conference on Intelligent Biology and Medicine (ICIBM) 2014: Genomics. The full contents of the supplement are available online at http://www.biomedcentral.com/bmcgenomics/supplements/16/S7.
