# Supplementary figures and images for: Dysregulated transcription across diverse cancer types reveals the importance of RNA-binding protein in carcinogenesis
Source: BMC Genomics. 2015 Jun 11;16(Suppl 7):S5. doi: 10.1186/1471-2164-16-S7-S5 (PMC4474540; doi:10.1186/1471-2164-16-S7-S5)

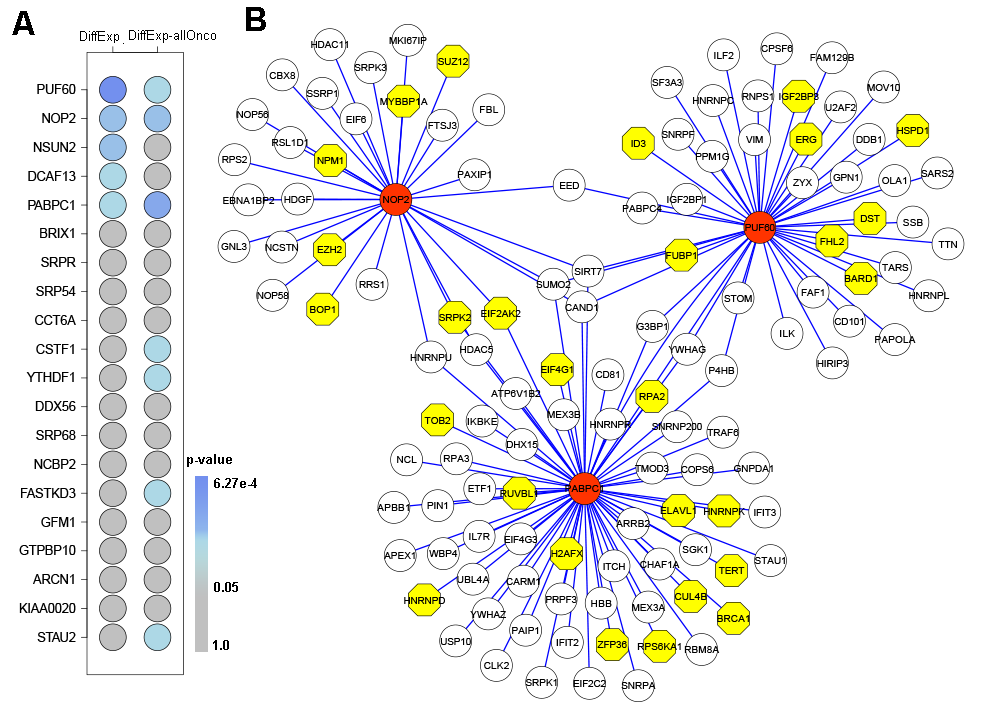

Supplement: Additional file 1 — Network of RBP-target interactions in LUAD. (A) Enrichment level of differentially expressed genes and allOnco in the targets of top 20 RBPs with highest fold change in LUAD. (B) PPI network comprising interactions between RBPs and differentially expressed targets in LUAD. The RBPs are those in top 20 and both differentially expressed genes and allOnco enriched in their interaction targets. RBPs are color coded as red, and their target allOnco are color coded as yellow. [file 1471-2164-16-S7-S5-S1.tif]

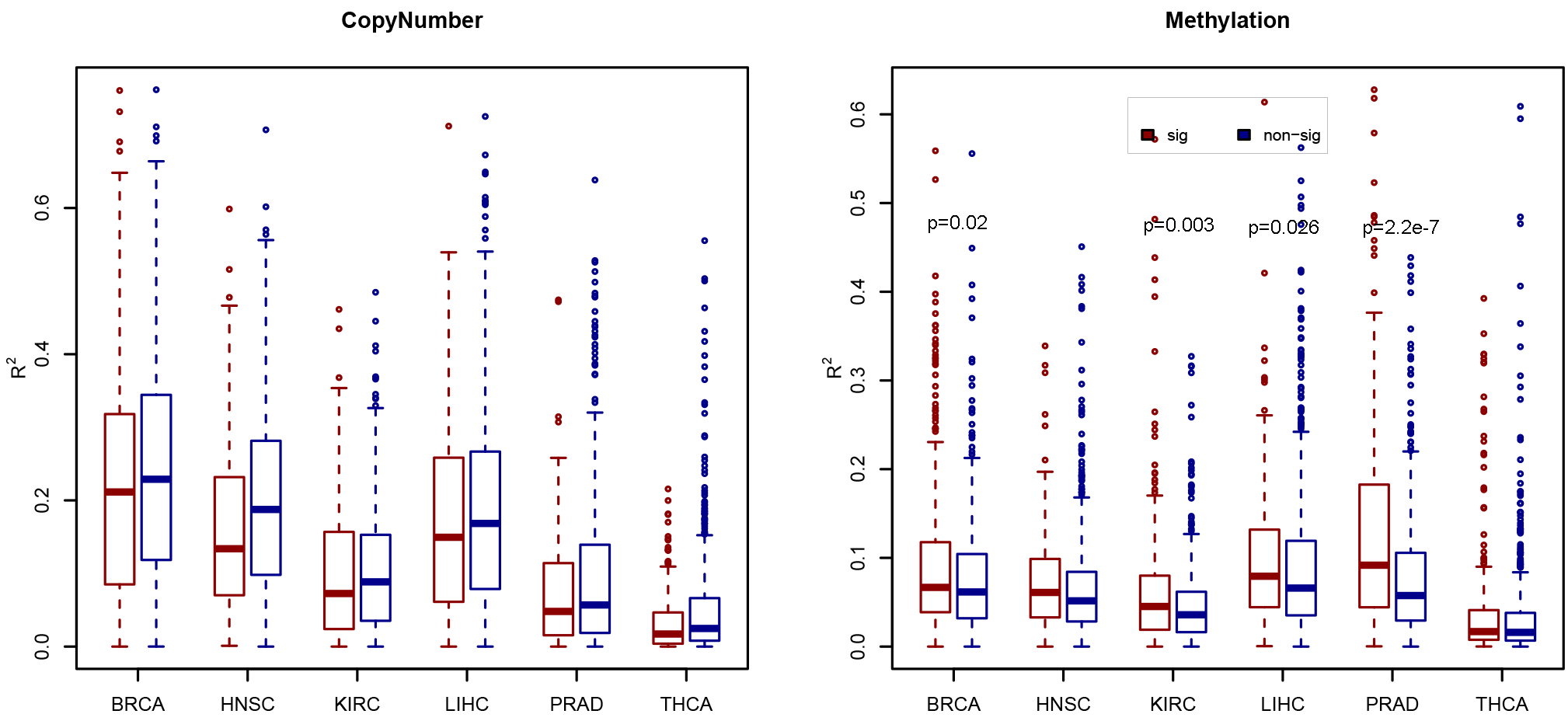

Supplement: Additional file 2 — Genetic and epigenetic alterations regulating RBPs. For the differentially expressed and other RBPs in cancer types of BRAD, HNSC, KIRC, LIHC, PRAD and THCA, we estimated the extent to which changes in copy number and methylation could explain the variation in their expression (R2). [file 1471-2164-16-S7-S5-S2.tif]
